# The Psychology of Nutrition with Advancing Age: Focus on Food Neophobia

**DOI:** 10.3390/nu11010151

**Published:** 2019-01-12

**Authors:** Emmy van den Heuvel, Annie Newbury, Katherine M. Appleton

**Affiliations:** Research Centre for Behaviour Change, Department of Psychology, Faculty of Science and Technology, Bournemouth University, Poole BH12 5BB, UK; annienewbury@yahoo.co.uk (A.N.); k.appleton@bournemouth.ac.uk (K.M.A.)

**Keywords:** food neophobia, older adults, physical disadvantage

## Abstract

Many factors impact on eating behaviour and nutritional status in older adults. Strategies can be suggested to combat the impact of these factors, including the development of novel food products, but food neophobia (“the reluctance to eat and/or avoidance of novel foods”) may be a barrier to the acceptance of these foods/products. This work aimed to investigate associations between food neophobia, physical disadvantage, and demographic characteristics in adults over 55 years old. Cross-sectional data from 377 older adults was analysed for relationships between food neophobia scores and physical disadvantage (denture wearing, help with food shopping and/or preparing, and risk of sarcopenia), controlling for age group, gender, living status, education, and employment level. Initial analyses demonstrated higher food neophobia scores in association with denture wearing (*Beta* = 0.186, *p* = 0.001). However, when demographic characteristics were also considered, food neophobia scores were no longer related to denture wearing (*Beta* = 0.069, *p* = 0.226) but instead were related to a higher age, living alone, and a shorter education (smallest *Beta* = −0.104, *p* = 0.048). Food neophobia may thus act as a barrier to the consumption of novel foods/products in those who are of higher age, are living alone, and have a shorter education.

## 1. Introduction

The prevalence in the UK of malnutrition or being at risk of malnutrition is estimated to extend to over 3 million people, with the majority (93%) of those at risk of malnutrition living in the community [1]. It has also been reported that energy intake decreases by 25% from the age of 40 to 70 years old [2]. Moreover, nutritional requirements change as people get older [3,4,5]; thus, dietary intake that was previously adequate may no longer suffice in older age and could potentially lead to malnutrition.

Many factors that contribute to reduced energy intakes are considered. Appetite and sensory abilities are known to reduce with age, while physical disabilities that can impact eating, food preparation, and food procurement can increase with age [2,6,7,8]. The hedonic appeal and sensory characteristics of foods play an important role in food choice [2,7,8,9,10], and sensory impairments are known to strongly impact food intake [2,6,7,11]. Physiological changes in dentition, oral and chewing abilities, gut motility and function, as well as physical changes in manual dexterity, physical balance and strength can impact eating, food preparation, and food procurement [2,6,7,8]. Deteriorations in visual abilities and in food recognition can also impact food preferences [2,6,8,11]. The effort involved in food preparation and the perceived inconvenience of some foods are also likely to increase [2,6,7,8]. Medical conditions and the use of medication often increase with aging and can impact eating behaviours and digestion [2,6,7]. There may also be more psychological factors that influence food choice, including health implications and issues such as ethical concerns, body-weight concerns, and concerns for the consumption of natural foods [8,9]. Other psychological factors may also increase with age, such as bereavement, depression, or dementia [2,6,7,8,9,10]. More social determinants of reduced food intake in older adults also include financial concerns and the cost of foods, living and eating alone, other aspects of social isolation, and the reduced ambiance of many eating environments [2,6,7,8]. 

Solutions to many of these issues are possible. Liking and tastiness can be increased by using complementary condiments and flavoured items [12,13,14,15,16,17,18]. The use of pre-cut or minced meat, slow-cooked meat, or softer products such as pâtes, eggs, and some dairy products may aid consumption in those with physical disabilities [19,20,21]. Slow-cooking and various forms of processing can also soften texture and aid consumption [22], and the use of pre-prepared, pre-cooked, or easy-to-prepare-and-cook foods and food products, such as canned products, may aid perceptions of convenience and effort [23]. Practical strategies such as freezing foods or bulk-buying and sharing foods, and the use of foods with longer shelf-lives such as eggs and canned, cured, or smoked food products, have also been suggested [24,25]. 

One often-suggested solution for increasing energy intake is the provision of supplements [26,27,28]. Prescribing oral nutritional supplements (ONS) is widely recommended in the management of malnutrition [26,27,28], but ONS are often disliked, particularly by those who are more able [29,30]. Intakes of ONS can also be low, increasing waste and cost for limited benefit [29]. Another possible solution to promote nutrient intakes in older adults is through fortified foods (e.g., protein enriched foods). Fortified foods, however, may also not be acceptable to all older adults [31,32]. It has been suggested that the acceptance of unfamiliar foods can be low in older adults [33,34,35], and ONS and fortified foods can be unfamiliar to many community-dwelling older adults. 

The acceptance of unfamiliar or novel foods is captured by the concept of food neophobia. Food neophobia is defined as the “reluctance to eat and/or avoidance of novel foods” [36], and although mainly thought to affect children between two and six years old [37], high scores of food neophobia have also been reported in adults [38,39,40,41]. Furthermore, higher food neophobia scores in adults have been associated with lower vegetable consumption [42,43,44], lower dietary quality [44], and lower dietary variety [39,45,46], and therefore may influence health status. While the validated food neophobia scale by Pliner and Hobden [36] refers to unfamiliar foods in general or ethnic foods as “novel foods”, food neophobia scores have been related to the reluctance to try different types of “novel foods”, including functional foods, organic foods, nutritionally modified foods, and genetically modified foods [38,39,47,48].

Oral nutritional supplements and fortified foods may be comparable to functional foods, and food neophobia scores have been related to a reluctance to try novel functional foods in adults, including older adults [41,49,50]. Urala and Lähteenmäki find negative correlations between food neophobia scores and willingness to use functional foods [49]. Stratton et al. reported that older adults with higher food neophobia scores were less willing to try a new functional food and show greater barriers to functional food consumption [41], and Siegrist et al. demonstrated that food neophobia scores predict willingness to buy functional foods in certain carriers [50].

Food neophobia can thus act as a barrier to the consumption of novel foods and could act as a barrier to the consumption of novel fortified foods or foods that have been specially developed for older adults. Such an association may impact negatively on an individual’s health status and could increase the risk of malnutrition. The negative impacts of this association will furthermore be compounded where older adults are already at increased risk of malnutrition. This work aimed to investigate the association between food neophobia and physical disadvantage, as a measure of increased risk of malnutrition, in older adults. 

## 2. Materials and Methods 

### 2.1. Participants

A possible association was investigated in complete cross-sectional questionnaire data from 377 participants from three different studies. Data from 216 participants were gained from a postal questionnaire study investigating the challenges and facilitators associated with egg consumption in a population-wide sample of older adults [51], data from 97 participants were gained from assessments taken at baseline for a randomised controlled trial investigating the impact of high-protein egg-based recipes and single-use herb/spice packets on egg and protein intakes [52], and data from 64 participants were collected specifically for these analyses. All participants were adults aged 55 years and over, living in their own homes in the UK at the time of questionnaire completion. Ethical approval for all data collection was granted by the Research Ethics Committee of Bournemouth University, prior to the commencement of each study.

### 2.2. Food Neophobia

Food neophobia in all studies was assessed using the validated 10-item “Food Neophobia Scale” [36]. The questionnaire requested agreement or disagreement with several statements, using the response categories “strongly disagree”, “disagree”, “neither agree nor disagree”, “agree”, and “strongly agree”. Responses were provided on a 5-point scale and scored from 1 to 5, respectively; therefore, the potential range of food neophobia scores was 10–50, where a higher score denotes a higher food neophobia and thus a higher reluctance to try new foods. Whereas the original “Food Neophobia Scale” has a 7-point response scale, a 5-point scale was chosen to match other measurement scales used within our studies. The 5-point scale has previously been accepted for use by others and has been related to willingness to try novel foods in adults [53,54,55,56].

### 2.3. Physical Disadvantage

Physical disadvantage was assessed using: (1) a binary measure of “at risk”/“not at risk” of sarcopenia based on the SARC-F questionnaire [57]; (2) a measure of the frequency with which individuals received help with food shopping or preparation; and (3) a measure of denture wearing. For 161 participants, the validated five-item SARC-F questionnaire [57] was used to assess symptoms of sarcopenia, whereas for 216 participants, an adapted version of the SARC-F was used, combining all questions. All responses were converted to a binary outcome of either “at risk” or “not at risk” following the instructions by Malmstrom and Morley [57]. Frequency of receiving help with food shopping or preparation was requested on a three-point scale—“never”, “sometimes”, “often”—scored 1–3, respectively, and responses were combined, as a direct measure of physical disadvantage of relevance to eating behaviour where higher scores reflect greater disadvantage. Denture wearing was requested also on a three-point scale—“no”, “partial dentures” or “full denture”—again scored 1–3, respectively, where higher scores reflect greater disadvantage. Denture wearing is directly related to eating abilities and has been associated with greater risk of frailty and malnutrition [58,59]. All participants were also asked about the frequency with which they ate out or away from home and had food delivered, but these questions were thought to be too ambiguous for use as measures of physical disadvantage. Positive answers to these questions could represent physical disadvantage (e.g., through use of services, such as “meals on wheels” or luncheon clubs), or lifestyles and preferences (e.g., through restaurant visits).

### 2.4. Demographic Characteristics

Demographic characteristics were also included in analyses of associations as possible confounders. Age, gender, education, and living status have been related to physical disadvantage [60,61] and food neophobia [36,39,43,55,62,63,64], and may therefore affect associations between physical disadvantage and food neophobia in this population. These questions requested: gender, age group or date of birth (converted to age group), living status (alone, with others), years of education, and most recent level of employment (unemployed, manual worker, non-manual worker, professional/management). Data on marital status were also collected, but these data were not included in analyses, because living status and marital status were highly correlated (*r* = −0.741, *p* < 0.001).

### 2.5. Analyses

Data analyses were performed using IBM SPSS Statistics software (version 19.0, Armonk, NY, USA). Chi square tests were first conducted to examine whether the sample was representative of the UK population over 55 years old according to the UK Census 2011 [65], in terms of age group and gender. Internal consistency was measured using Cronbach’s alpha. Mean food neophobia scores, standard deviations (SD), and ranges were generated for different subgroups based on demographic and lifestyle characteristics. Additionally, Pearson correlations (*r*) and point-biserial correlations (*r*_pb_) were assessed between all measures that would be included in the regression. Hierarchical multiple linear regression analyses were then conducted to assess whether factors related to physical disadvantage were associated with food neophobia scores, controlling for several demographic characteristics. Model 1 comprised denture wearing, help with food shopping and/or preparing, and risk of sarcopenia to predict food neophobia scores. Model 2 comprised model 1 plus age group, gender, living status, education, and employment level. 

## 3. Results

### 3.1. Participants

Participant characteristics for all 377 participants are given in Table 1. The sample was representative of the UK older population in terms of gender (χ^2^ (2) = 0.63, *p* > 0.05), according to the UK Census 2011, but not in terms of age (χ^2^ (6) = 84.75, *p* < 0.01). 

Internal consistency for the food neophobia scale items, measured as Cronbach’s alpha, was 0.87.

### 3.2. Correlations

Table 1 shows the food neophobia scores (Mean + SD, range) for different groups with a specific demographic characteristic. Food neophobia scores were significantly correlated with age (*r* = 0.222, *p* < 0.001), living alone (compared to living with others) (*r_ph_* = −0.188, *p* < 0.001), shorter education (*r* = −0.288, *p* < 0.001), lower employment levels (*r* = −0.212, *p* < 0.001), denture wearing (*r* = 0.225, *p* < 0.001), receiving help with food shopping and/or preparing (*r* = 0.138, *p* = 0.007), and being at risk of sarcopenia (*r_ph_* = 0.166, *p* = 0.001). There was no correlation between food neophobia and gender (*r_ph_* = −0.043, *p* = 0.407). Denture wearing, help with food shopping and/or preparing, and risk of sarcopenia were significantly correlated with each other (largest *r* = 0.553, *p* < 0.001). Each measure of physical disadvantage was also significantly correlated with higher age group, female gender, shorter education, and lower employment level (largest *r* = 0.424, *p* < 0.001). Wearing dentures and risk of sarcopenia were also significantly correlated with living alone (compared to living with others), and the correlation for receiving help with food shopping and/or preparing approached significance (*r* = −0.098, *p* = 0.057). Higher age group was also significantly correlated with living alone and shorter education; male gender was significantly correlated with living with others, longer education, and higher employment level; and a longer education was significantly correlated with living with others and a higher employment level (largest *r* = 0.452, *p* < 0.001). No evidence of multicollinearity was found for the variables included in the regression (largest *r* = 0.553, *p* < 0.001).

### 3.3. Regression Analyses

#### 3.3.1. Model 1: Physical Disadvantage Predicting Food Neophobia

The multiple linear regression model including denture wearing, help with food shopping and/or preparing, and risk of sarcopenia significantly predicted food neophobia scores (*R* = 0.248, *R*^2^ = 0.061, adjusted *R*^2^ = 0.054, F(3373) = 8.132, *p* < 0.001). Higher food neophobia scores were significantly associated with denture wearing (*Beta* = 0.186, *p* = 0.001). All *Beta* values and *p* values can be found in Table 2.

#### 3.3.2. Model 2: Physical Disadvantage and All Demographic Characteristics Predicting Food Neophobia

Model 2 also significantly predicted food neophobia (*R* = 0.372, *R*^2^ = 0.139, adjusted *R*^2^ = 0.120, F(8368) = 7.407, *p* < 0.001). The association between food neophobia and denture wearing reduced and was no longer significant (*Beta* = 0.069, *p* = 0.226), whereas associations were found between higher food neophobia scores and a higher age group (*Beta* = 0.112, *p* = 0.043), living alone (*Beta* = −0.104, *p* = 0.048), and a shorter education (*Beta* = −0.185, *p* = 0.001). All *Beta* values and *p* values can be found in Table 2.

## 4. Discussion

This work aimed to investigate the association between physical disadvantage and the reluctance to try novel foods in adults over 55 years old. Food neophobia scores were high compared to those found in other studies [55], suggesting a high reluctance to try novel foods in this population. Initial analyses showed that denture wearing was associated with higher food neophobia scores, or a higher reluctance to try novel foods, but when demographic characteristics were also considered, this relationship disappeared, and food neophobia was instead related to a higher age, living alone, and shorter education.

A positive association between denture wearing and food neophobia was found in the initial analyses. Dental status is known to affect food choice and nutritional status in older adults [66,67]. Edentate elderly people are known for not wanting to eat the foods they find difficult to eat [66], and difficulties with chewing have been related to food choice (e.g., avoiding foods that are difficult to eat) and nutrient intake (e.g., lower total protein intake) [22,68,69,70,71]. Additionally, eating difficulties as well as food pickiness have been associated with risk of malnutrition in older adults [72]. Our findings suggest that oral deficiencies, as denoted by denture wearing, may not only affect known food choice but also result in a greater reluctance to try new foods. It is plausible that individuals with eating difficulties may be more wary of novel foods or foods with unknown contents and textures. These findings may have implications. If the older adults who are at increased nutritional risk also tend to be more reluctant to try novel foods, this reluctance could be an important barrier in introducing (novel/unfamiliar) ONS or fortified foods to those who need them most. Novelty may be a critical factor to consider when designing food products that are acceptable to older adults with physical disadvantages; thus, the provision of ONS or fortified foods that are similar to well-known and habitually eaten foods may have a clear advantage. With the high prevalence of malnutrition and the need to change eating behaviour in the older population, a better understanding of the willingness to try novel foods may facilitate the treatment of malnutrition or help to change eating behaviour in at-risk populations. Importantly, however, the association in our data was only found in denture wearing as a measure of physical disadvantage, while other measures of disadvantage were less important. These findings suggest that concerns in relation to novel foods may only apply to those with disadvantages or disabilities that are specific to eating, as opposed to disadvantages that are more general or that affect other aspects of daily life [27].

However, when demographic characteristics were also considered, the relationship between denture wearing and food neophobia disappeared, and food neophobia was instead related to a higher age, living alone, and a shorter education. These findings suggest that the relationship between denture wearing and food neophobia is at least in part a result of high levels of denture wearing in individuals who are older, are living alone, and/or experienced a shorter education. A positive association between food neophobia and age is in line with other research that demonstrates increasing food neophobia with age when considering wide age ranges of adults [39,43,55,62]. These effects are plausibly explained as a result of changes to the food environment with time. The food environment has changed over time, resulting in an environment that is now significantly more varied and diverse and includes a substantially higher number of foreign, ethnic, and unusual foods than were available just 20 years ago. Different age groups have grown familiar with different food environments [37], and a greater familiarity with a higher number of different foods has been related to lower food neophobia [39,45,46]. Alternatively, a higher food neophobia with increasing age may be explained as a result of increased frailty or a perceived increased susceptibility to illness as a result of foreign food bodies [37]. An explanation based on frailty would predict an association between food neophobia and physical disadvantage, which was not found, but there may be a valuable distinction to be made here between actual physical disadvantage and perceived physical disadvantage. Our study measured actual physical disadvantage based on physical abilities, and a measure of perceived disadvantage may have been more appropriate. It has previously been suggested that not all those who need help receive it and that not all those who receive help need it [73]. Our measures of physical disadvantage may also have been inadequate, as only limited aspects of physical disadvantage were measured. Moreover, not all studies demonstrate an association between age and food neophobia in adults [38,41,45], and some studies have demonstrated a decrease in food neophobia with age [36,63,64]. However, these studies can suffer from limited numbers at higher age ranges [36,38,63,64], while food neophobia has been shown to increase mainly in the age groups from 49, 55, or 66 years old [39,55,62].

A lower exposure to more varied and more diverse food environments may also explain the relationships found here between higher food neophobia scores, living alone, and a shorter education. It is plausible that living alone can result in more restricted food choices and eating opportunities and exposure to a more limited variety of foods that may again explain higher food neophobia scores [39,45,46]. It has been reported that older adults living alone, especially older men, show lower food diversity, decreased energy intake, and poorer diet quality [74,75,76,77,78]. The food neophobia scores of people living together tend to be similar, e.g., families with children [46], an effect that may also be related to a similar exposure to novel foods and a variety of different foods, but differences in food neophobia scores between those living alone and those living with others in absolute terms have not previously been reported.

It is also plausible that a shorter formal education may be associated with more limited food choices and food environments, through a lower confidence with cooking techniques and a lower reliance on different sources when learning to cook [79] and through associations with aspects of socio-economic status, such as opportunities for travel and eating out. Higher education alongside higher socio-economic status, has been associated with higher diet quality and dietary variety and a lower risk of malnutrition [80,81,82]. Associations between education and food neophobia, however, may also be associated more directly with lower nutritional knowledge [83]. Lower nutritional knowledge has previously been related to lower diet quality [84,85] and lower willingness to try novel functional foods [86]. Previous research also shows a negative relationship between education and food neophobia in studies with wide age ranges [38,39,43,55,47], although not all studies show this relationship [41,45]. Studies have shown that providing positive information about the health benefits of a novel food can affect willingness to try the food, suggesting a role for knowledge and information [87,88,89].

In our final regression model, no associations were found between food neophobia scores and gender or employment level. The results for gender are consistent with previous research [36,38,41,45,55,62,63]. A small number of studies show that adult males have higher food neophobia scores than females [39,43], and these results may again be related to exposure to and familiarity with a greater variety of foods, especially where the women may more often be responsible for food shopping and preparation [43]. Gender differences in willingness to try novel foods may also depend on the type of food, because gender has been shown to predict intake of some but not other fortified food products [90]. Associations between employment level and food neophobia have also not been previously reported. Other research, however, has shown a negative relation between income and food neophobia [41,43,55]. We assessed employment level, as income can be a sensitive question to research participants [91], but this question is potentially not always related to the income levels of those who are retired.

The findings in relation to age, living status, and education also have important implications. Nutritional requirements change with age and eating habits often do not change accordingly [2,6,7]. Eating behaviour is strongly influenced by past behaviour, upbringing, and habits, and changes to these habits can be difficult [9,10]; therefore, it is important for future research to focus on facilitating changing eating behaviour in older adults to match the change in nutritional requirements. Our findings suggest that interventions using novel food products should consider food neophobia as a potential barrier to acceptance, especially when targeting older adults who are older, living alone, and less educated. An important aspect here might be the perceived novelty of a food product, and future research into perceptions of novelty may be of value. New combinations of familiar foods, for example, or fortified foods that are similar to familiar foods may be more acceptable than completely novel food products. Some studies have shown greater acceptance for novel foods that are similar to familiar foods [47,87,92]. The British Association for Parenteral and Enteral Nutrition (BAPEN) also recommends a “food first” approach [93], and it has been shown that enriching foods with known foods (e.g., cream or cheese) and adding known tastes and flavours can increase food intake in older adults [12,13,14,15,18]. Other studies have significantly increased protein and energy intakes in hospital patients by combining conventional foods and supplementation [94,95]. Future research could also examine approaches to overcome the reluctance to try novel foods in older adults. Lack of awareness of the health benefits of ONS or fortified foods may be a barrier to using these foods [49,96], and the provision of information has previously been related to the willingness to try a novel food [87,88,89]. However, the effect of information may depend on individual preferences and beliefs [97], and many older adults may be sceptical and/or may not be open to nutritional advice [25,35,98].

Strengths of this work include our sole focus on adults over 55 years old and the large sample size. Within this sample however, there were limited numbers of individuals with high physical disadvantage, and the sample shows relatively high employment levels and long education, possibly due to volunteer bias. The analysis is also restricted by our limited measures of physical disadvantage—we have no measures of perceived disadvantage, and some aspects of disadvantage, e.g., reduced sensory abilities and reduced chewing abilities, were also not assessed [64,99,100]. Reduced abilities may also occur in the absence of any disadvantage, and we made no assessment of observed changes to these abilities. Moreover, there was no measure of dietary intake, physical activity, or medical conditions and prescribed diets, and these factors may be related to physical disadvantage and affect eating behaviour and therefore could be important to consider. We also did not assess specific reluctance to try ONS or fortified foods, and future research might explore whether these food products are affected by food neophobia. Denture wearing was assumed to represent poorer oral health and eating abilities, but it is possible that older adults who do not wear dentures and retain few natural teeth or have bad oral health, may have more problems with biting and chewing than those with dentures [101].

## 5. Conclusions

In initial analyses, food neophobia was significantly associated with denture wearing, but these findings disappeared when demographic variables were also considered, and food neophobia was instead associated with a higher age, living alone, and a shorter education. These findings suggest that older adults with higher age, living alone and shorter education may show a greater reluctance to try novel foods. Food neophobia thus may act as a barrier to the consumption of novel supplements or fortified foods in these groups. Consequently, treatments for age-related conditions such as malnutrition that focus on novel foods need to be carefully designed.

## Figures and Tables

**Table 1:** Participant characteristics (*N* = 377).

	N (%) or Mean ± SD	Food Neophobia (Mean ± SD)	Range of Food Neophobia Scores	Pearson (*r*)/Point-biserial (*r_pb_*) Correlation with Food Neophobia
Age				*r* = 0.222 *
55–59 years old	38 (10.1%)	22.9 ± 6.4	10–40	
60–64 years old	55 (14.6%)	23.5 ± 6.8	10–41	
65–69 years old	84 (22.3%)	22.5 ± 6.7	10–38	
70–74 years old	90 (23.9%)	25.4 ± 7.2	11–49	
75–79 years old	60 (15.9%)	25.2 ± 7.2	10–43	
80+ years old	50 (13.3%)	28.4 ± 7.2	14–49	
Gender				*r_pb_* = −0.043
Female	197 (52.3%)	24.9 ± 7.4	10–49	
Male	180 (47.7%)	24.3 ± 6.9	10–43	
Living status				*r_pb_* = −0.188 *
Alone	127 (33.7%)	26.5 ± 7.5	11–49	
With others	250 (66.3%)	23.6 ± 6.8	10–41	
Education in years (Mean + SD, Range)	14 ± 3, 7–23			*r* = −0.288 *
Most recent employment level				*r* = −0.212 *
Unemployed	15 (4.0%)	26.5 ± 5.6	14–36	
Manual worker	80 (21.2%)	27.1 ± 7.8	10–49	
Non-manual worker	124 (32.9%)	24.8 ± 6.4	10–42	
Professional/Management	158 (41.9%)	23.0 ± 7.2	10–49	
Denture wearing				*r* = 0.225 *
No	238 (63.1%)	23.5 ± 6.8	10–43	
Partial dentures	105 (27.9%)	25.8 ± 7.2	11–49	
Full dentures	34 (9.0%)	28.5 ± 7.9	13–49	
Receiving help with food shopping or preparing				*r* = 0.138 **
Never	317 (84.1%)	24.1 ± 7.3	10–49	
Sometimes	38 (10.1%)	27.4 ± 5.7	16– 41	
Often	22 (5.8%)	26.7 ± 6.9	13–36	
Risk of sarcopenia (SARC-F)				*r_pb_* = 0.166 **
Not at risk	332 (88.1%)	24.2 ± 7.0	10–49	
At risk	45 (11.9%)	27.8 ± 7.7	13–49	

*p < 0.001, **p < 0.01.

**Table 2:** Outcomes of the multiple linear regression model assessing the effect of physical disadvantage and demographic characteristics on food neophobia. Significant effects are given in bold (*p* < 0.05).

		*Beta*	*p* value
Model 1	**Denture wearing**	**0.186**	**0.001**
	Help with food shopping and/or preparing	0.046	0.452
	Risk of sarcopenia	0.080	0.199
Model 2	Denture wearing	0.069	0.226
	Help with food shopping and/or preparing	0.030	0.613
	Risk of sarcopenia	0.017	0.785
	**Age group**	**0.112**	**0.043**
	Gender	0.032	0.518
	**Living status**	**−0.104**	**0.048**
	**Education**	**−0.185**	**0.001**
	Employment level	−0.094	0.092
